# Supplementary material for: Environmental Enrichment Induces Epigenomic and Genome Organization Changes Relevant for Cognition
Source: Front Mol Neurosci. 2021 May 5;14:664912. doi: 10.3389/fnmol.2021.664912 (PMC8131874; doi:10.3389/fnmol.2021.664912)
Supplement: Supplementary file 1 [file Table_1.DOCX]

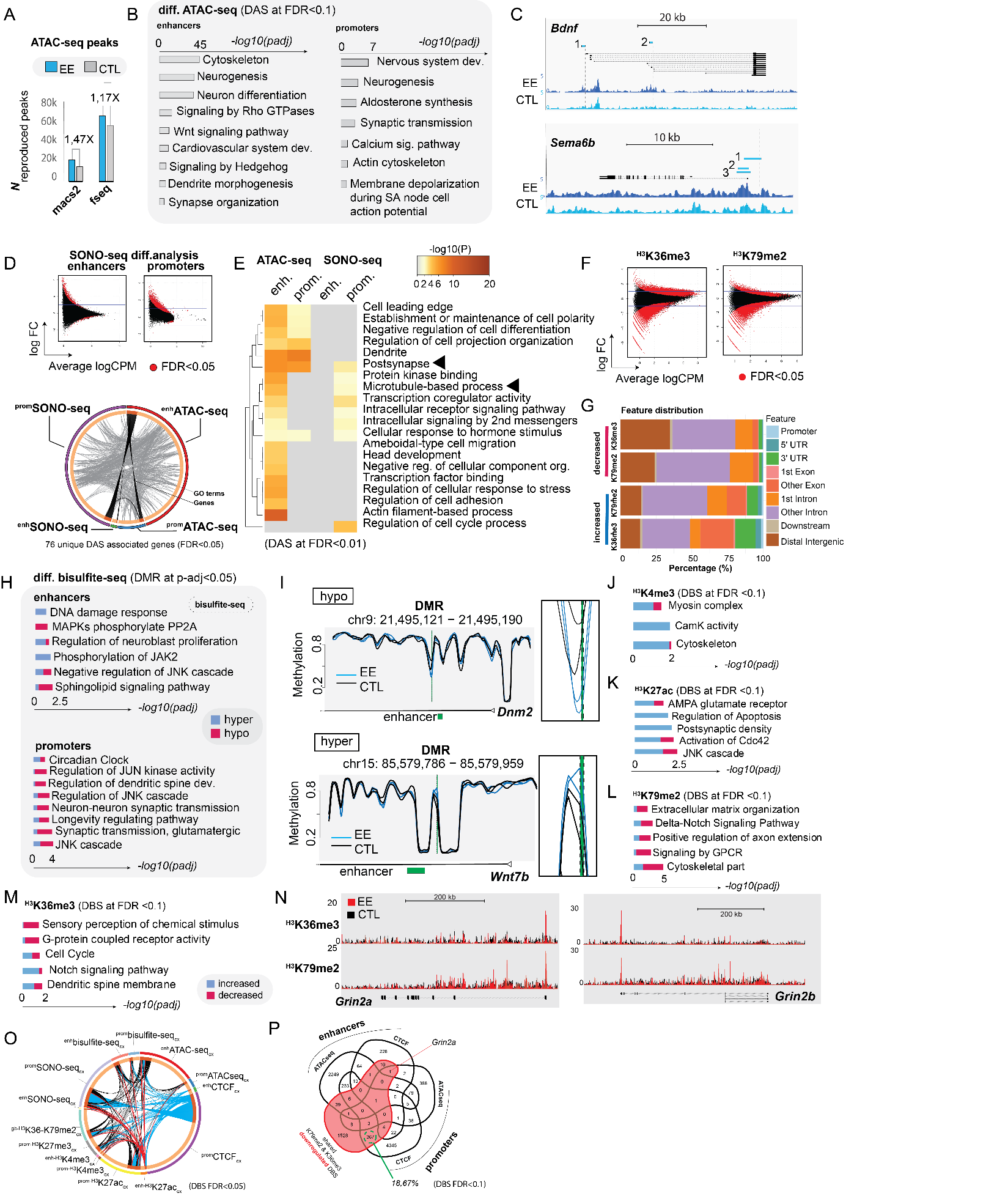
**Figure S1**

**Figure S1. Chromatin accessibility and epigenetic changes induce by EE in cortex homogenate.** ATAC-seq, SONO-seq, bisulfite-seq and histone marks results at postnatal day [P51]. **A)** Enrichment of EE and CTL samples over a consensus peak-dataset computed using both macs2 and fseq. Bar-plot shows that the number of peaks found by macs2 and fseq was greater in EE compared to CTL samples. **B)** Gene ontology analysis of previous regulatory regions: enhancers (left bar-plot) and promoters (right) (Table S2, p-value < 0.05 – Bonferroni adjusted). **C)** Cortex homogenate UCSC screenshot examples of ATAC-seq differential accessibility sites at P51, showing increased accessibility in the promoter regions of *Bdnf* and *Sema6a* genes. Numbers 1 and 2 correspond to fseq/macs2 peak calls and number 3 to the defined promoter region (Method Details). **D)** MA-plot showing the log(fold change) vs log(counts per million) of differential SONO-seq signal on enhancers (left) and promoters (right) at P51 due EE (red dots are significant sites at FDR<0.05, Table S2, Method Details). Circos plot show the intersection of SONOseq and ATAC-seq differential results due EE at FDR<0.05. **E)** Metascape gene ontology analysis of differential enhancer associated genes and promoters in both ATAC-seq and SONO-seq at FDR<0.01. **F)** Differential analysis of H3K36me3 (left MA-plot) and H3K79me2 (right) on enhancer regions (red dots denote significant results at FDR<0.05. **G)** Genome distribution plot of enhancers showing differential activity of H3K36me3 and H3K79me2 due EE. Around 15-25% of these regions are distal intergenic enhancers. **H**) GO analysis (at p-value < 0.05 Benjamini-Hochberg adjusted) of bisulfite sequencing differential analysis of enhancers (upper bar-plot) and promoters (bellow). **I)** Selected examples of hypo (upper plot) and hyper methylation regions (bellow). **J, K, L, M)** GO analysis of H3K4me3 (K), H3K27ac (L), H3K79me2 (M) and H3K36me3 (N) p-value < 0.05 Benjamini-Hochberg adjusted**. N)** UCSC genome browser examples of glutamatergic genes showing decreased binding of H3K79me2 and H3K36me3 upon EE. **O)** Circos-plot showing the intersection of differential analysis in all cortical epigenetic marks upon EE at FDR<0.05. Highlighted in blue differential CTCF binding and in red H3K27ac. **P)** Venn-plot of potential impact into transcription-associated marks due differential activity shown in regulatory regions: enhancers and promoters. Associated-genes of ATAC-seq and CTCF showing corresponding differential enhancer and promoter activity intersected with consensus differential H3K79me2 & H3K36me regions (i.e. genes that shared differential gene-body activity due EE in both histone marks).

**Figure S2**

**
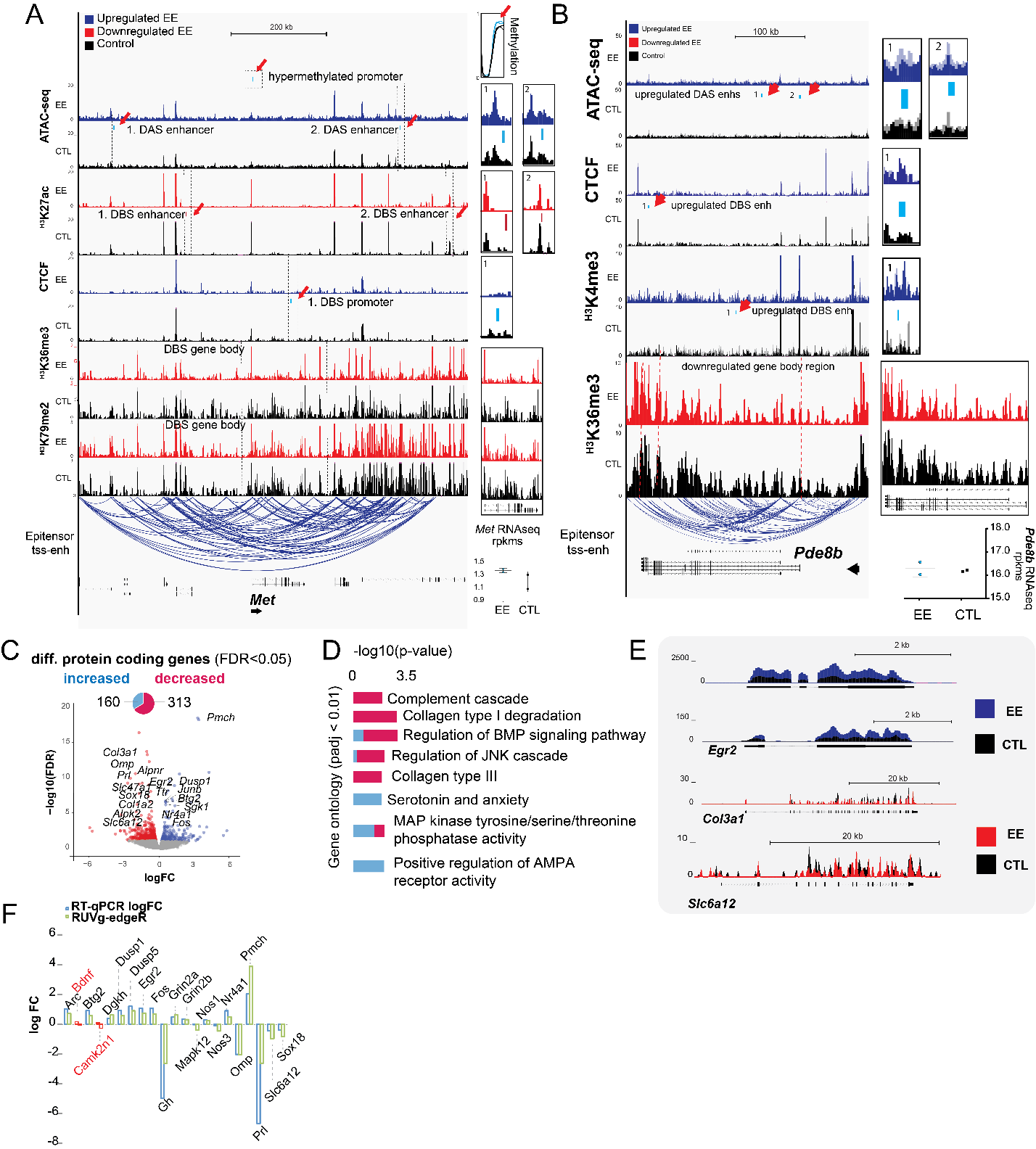
**

**Figure S2. Transcriptional and translational changes due EE. A, B)** *Met* and *Pde8b* USCS screenshot examples showing gene-body downregulation of H3K36me3 and H3K79me2 together with differential CTCF and ATAC-seq signals upon EE**. C)** Differential expression of poly-A expression volcano plot representing changes found using RUV-g correction method. Blue, upregulated genes; red, downregulated genes at FDR<0.05. **D)** Gene ontology analysis of selected significant (p-value < 0.01 Bejamini-Hochberg corrected) terms of differential expressed genes (DEG). Blue bars represent the percentage of the p-value enrichment of genes that are upregulated and pink the proportion of downregulated genes. **E)** Screenshot of genome browser DEG examples. **F)** poly-A validation plot by qPCR. Blue bars represent the logFC of EE vs CTL measured by qPCR and compared to DEG found in the differential analysis using RUV-g correction.

**Figure S3**


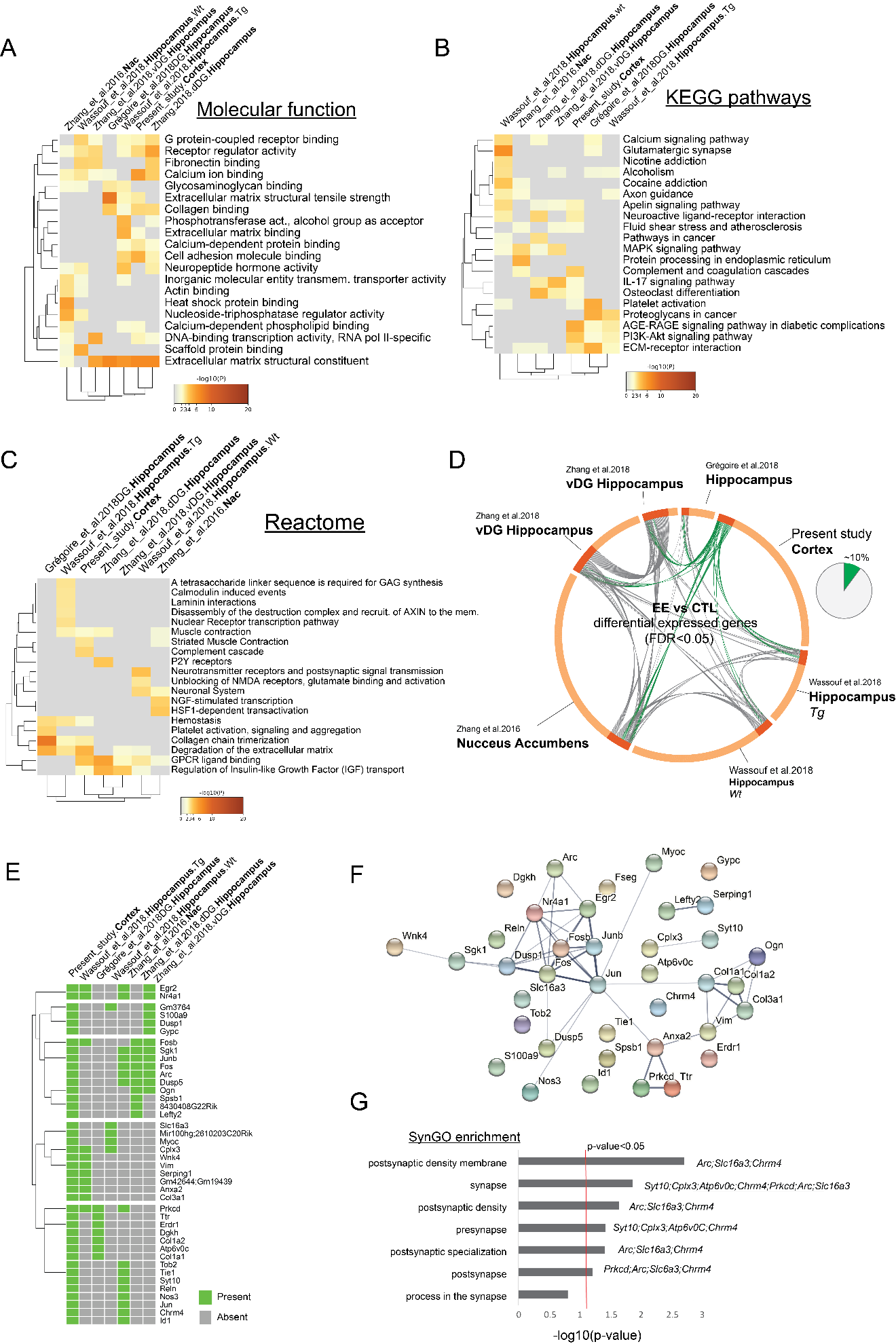


**Figure S3. Cross-comparison of published differential expressed genes due EE.** Cross-comparison of DEGs found in the present study with previous published studies **A,B, C)** Metascape molecular function (A), KEGG (B) and Reactome (C) enrichment (p-adj<0.05, min.overlap=3). **D)** Intersection of induced DEGs due EE (FDR<0.05). 10% of DEGs in the present study are present in other published analysis. **E)** Heatmap of DEGs that overlap with other studies showing the greatest consistency for *Arc* and *Nr4a1* genes. **F)** String db medium confidence of intersected DEGs showing a clear clustering of early expressed genes (left) and extracellular matrix components (right). **G)** SynGO enrichment of overlapped DEGs showing a clear enrichment in postsynaptic density membrane elements such as *Arc*, *Slc6a3* and *Chrm4*.

**Figure S4**

**
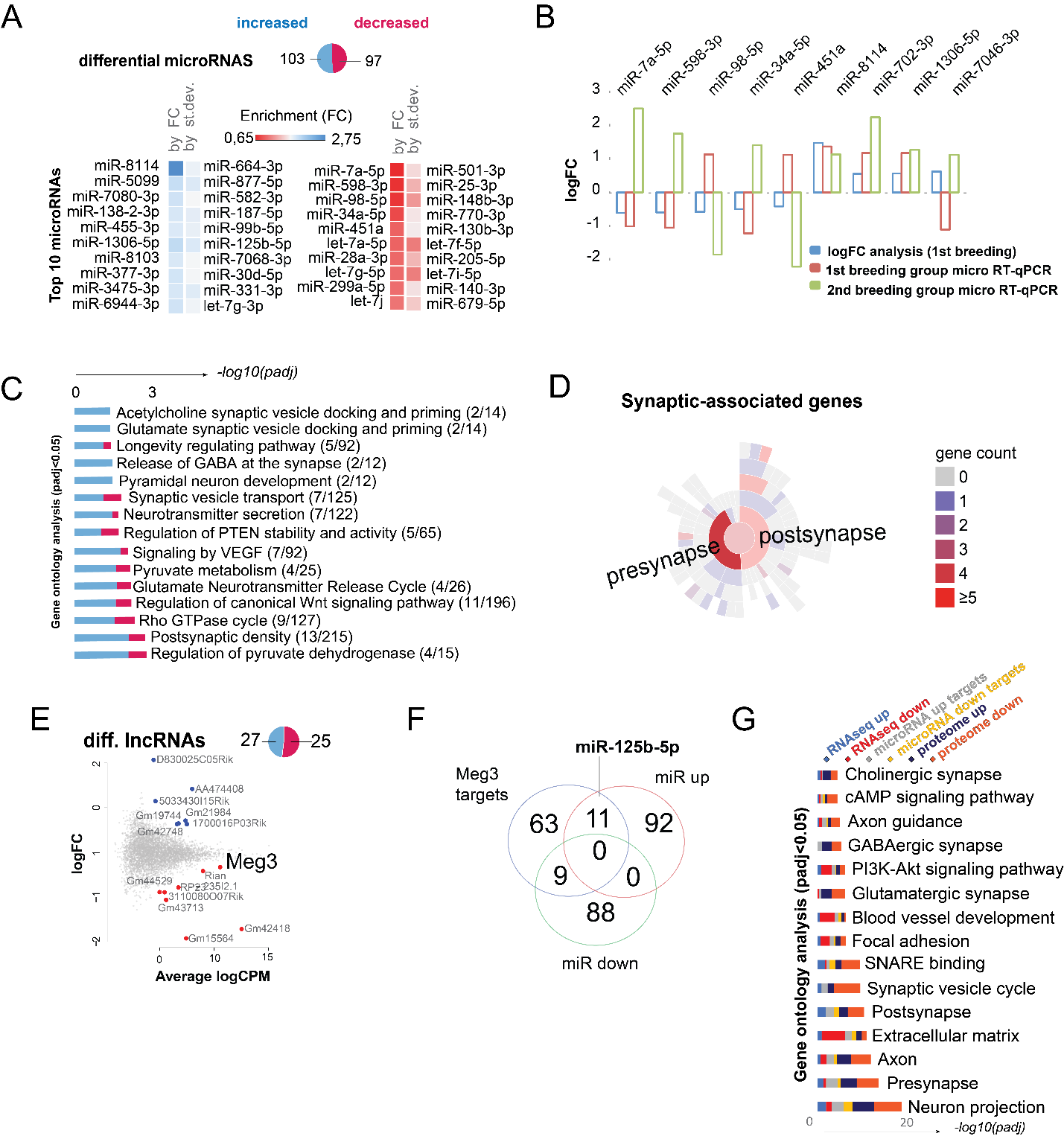
**

**Figure S4. Non-coding transcriptomic and proteomic changes due EE.
A)** Top differential upregulated (left) and downregulated (right) microRNAs sorted by fold change and p-value. Up-right, circle-plot of total number of differential microRNAs found in our data. **B)** Validation of microRNAs in different batch set of animals (second breeding) by qPCR (Method Details). **C)** microRNA targets gene ontology (p-adj <0.05). **D)** SynGo synapse enrichment analysis of microRNA targets. **E)** Differential expression of lncRNAs highlighting *Meg3.* **F)** Intersection of *Meg3* predicted microRNA binding with LncBase v2.0 with differential microRNAs found in our dataset (Table S4). **G)** Gene ontology analysis of significant terms (p-value < 0.05 adjusted) found in the differential analysis of RNAseq, microRNA and proteome.

**Figure S5**

**
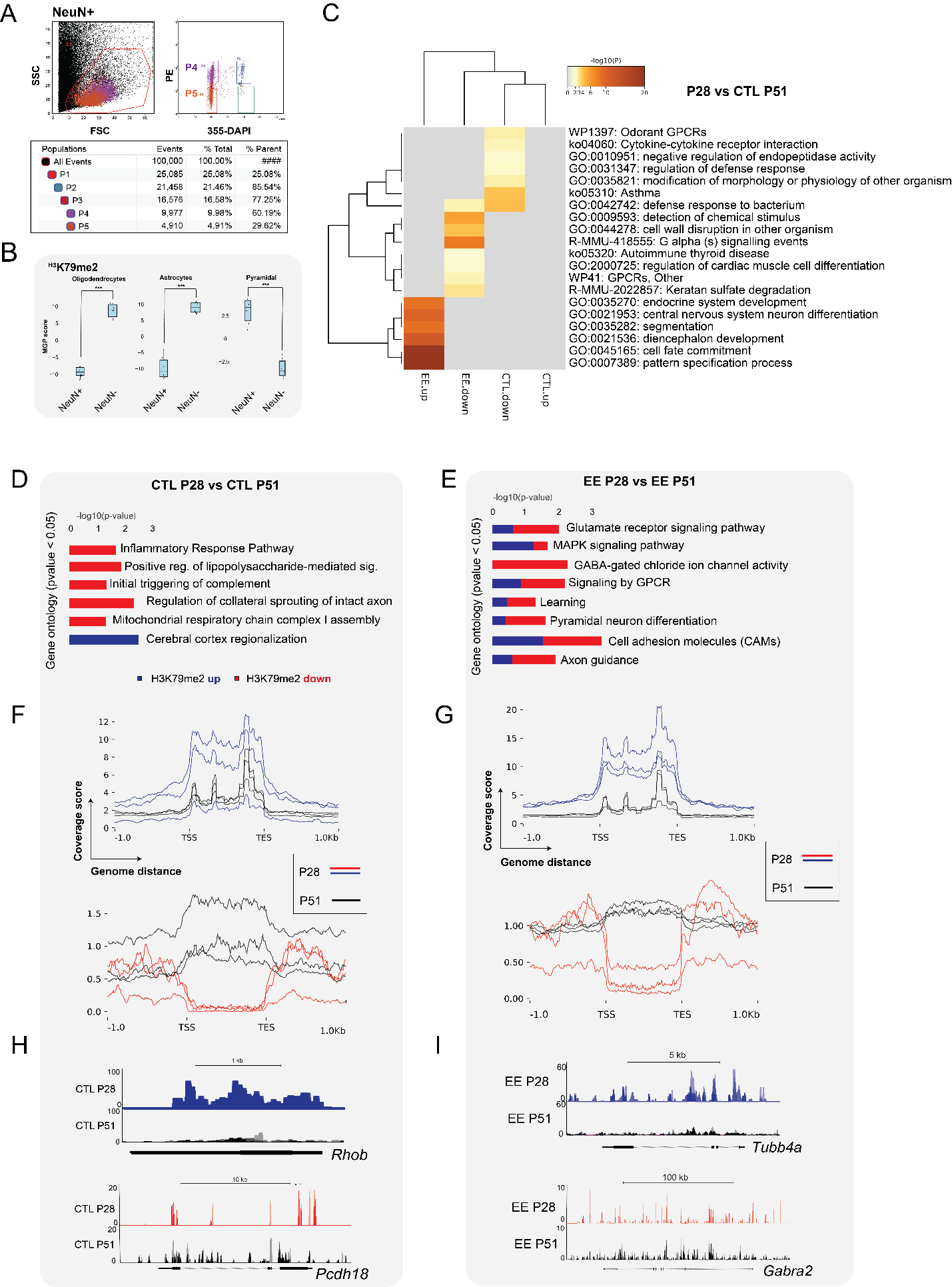
**

**Figure S5. H3K79me2 transcription-associated changes due EE in sorted populations. A)** Gating strategy: FAC-sorting report plots of extracted NeuN+ and NeuN- populations. Plot y-axis shows side-scattered light (SSC) versus x-axis Forward-scattered light (FSC) plot of NeuN+ nuclei preparation. The SSC value reports the complexity of the sample measured by the refraction of the laser light beamed in the surface of the nuclei or cells in the flow. The FSC reports the intensity of the signal given by the conjugated antibody (Alexa Fluor-555). Right**,** intensity plot of the signal of the conjugated antibody (PE, Alexa-555) versus DAPI intensity. P4 and P6 represent the nuclei positive for the neural marker NeuN+. **B)** Cell specificity of H3K79me2 NeuN+ and NeuN- populations using curated gene specific cell markers discovered by single-cell RNAseq (Mancarci et al., 2017) (Method Details). Neuronal and non-neuronal H3K79me2 counts in gene body regions were normalized to rpkms. The cell specify is assessed by the marker gene profile (MGP) score, and it was calculated for oligodendrocytes (left), astrocytes (center) and pyramidal neurons (right) markers. Significant oligodendrocytes and astrocytes (pvalue< 0.01) markers were found to be associated more to NeuN- populations, meanwhile significant pyramidal markers (pvalue<0.01) were found to be associated to NeuN+.**C)** Metascape gene ontology enrichment for P28 vs P51 in CTL and EE samples (p-adj <0.01) **D)** ClueGO gene ontology analysis of P28 vs P51 of CTL samples (p-adj <0.05). **E)** ClueGO gene ontology analysis of P28 vs P51 of EE samples (p-adj <0.05). **F, G)** Deeptools coverage plots of H3K79me2 differential regions (FDR<0.05). **H,I)** Genome browser captions of P28 vs P51 differential H3K79me2 associated genes in both CTL and EE samples.

**Figure S6**


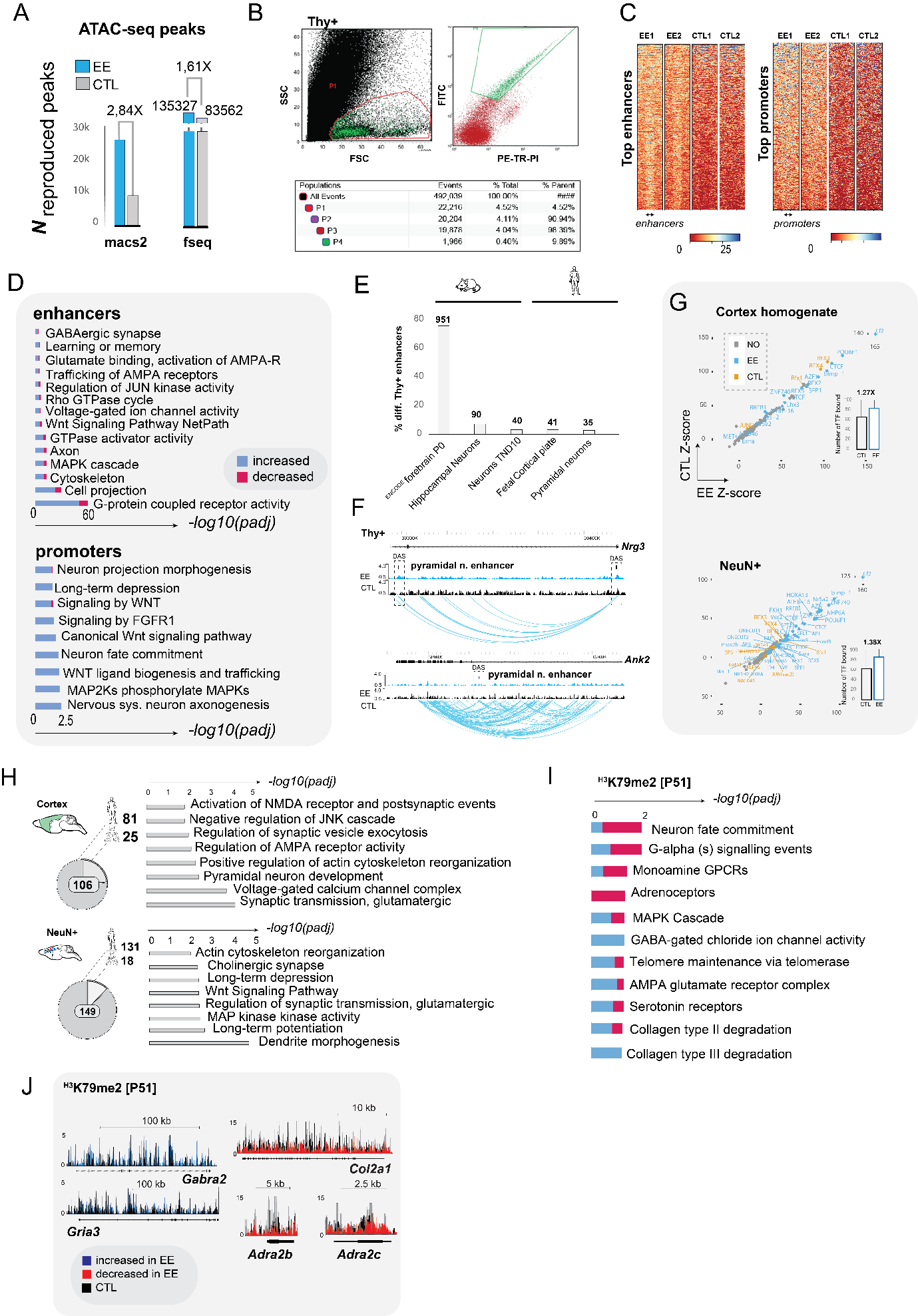


**Figure S6. Chromatin accessibility and transcription-associated changes due EE in sorted populations. A)** Enrichment of EE and CTL samples over a consensus peak-dataset computed using both macs2 and fseq in NeuN+ sorted nuclei. Bar-plot shows that the number of peaks found by macs2 and fseq was greater in EE compared to CTL samples. **B)** Side-scattered light (SSC) versus Forward-scattered ligh (FSC) plot of YFP+ pyramidal neurons preparation (Tg-Thy1+mice, left), and FITC signal of the YFP neuronal cells (right). **C)** Top changes of ATAC-seq in regulatory regions of Thy1+ pyramidal neurons (n=2 per condition). **D)** Gene ontology analysis of the Thy+ differential accessibility sites in enhancers (upper plot) and promoters (bellow). **E)** Thy+ enhancer intersection showing differential accessibility due EE with mouse ENCODE forebrain P0 enhancers (ENCFF711DUZ), hippocampal and TND10 mature neurons and lifted mouse to human intersection with human fetal cortical plate and pyramidal neuron enhancers (Dong et al., 2018; Fernandez-Albert et al., 2019; de la Torre-Ubieta et al., 2018; Thakurela et al., 2015) **F)** Epigenome WashU screenshot of shared enhancers with human pyramidal neurons lifted to mouse genomic coordinates. **G)** Footprint analysis. Z-scores regression plots showing transcription factors binding (control - orange, EE - blue), bar-plots represent the accumulated number of TF bound in both conditions in whole cortex (left) and NeuN+ (right). **H)** CTCF bound genes found for cortex (upper plot) and NeuN+ samples (bellow). Motifs detected were human and *Drosophila* specific. Drawings (up-right of the pie-plot) assigned the number instances found per specie. Next to it (right) associated gene ontology analysis of selected terms (pvalue < 0.05 Benjamini-Holchberg adjusted, Table S2). **I)** H3K79me2 gene ontology analysis of EE vs CTL at P51 (p-value < 0.05, Benjamini-Hochberg adjusted) **J**) NeuN+ H3K79me3 at P51 UCSC screenshots of increased (blue) and decreased (red) binding due EE stimulation versus CTL samples (black tracks).

**Figure S7**


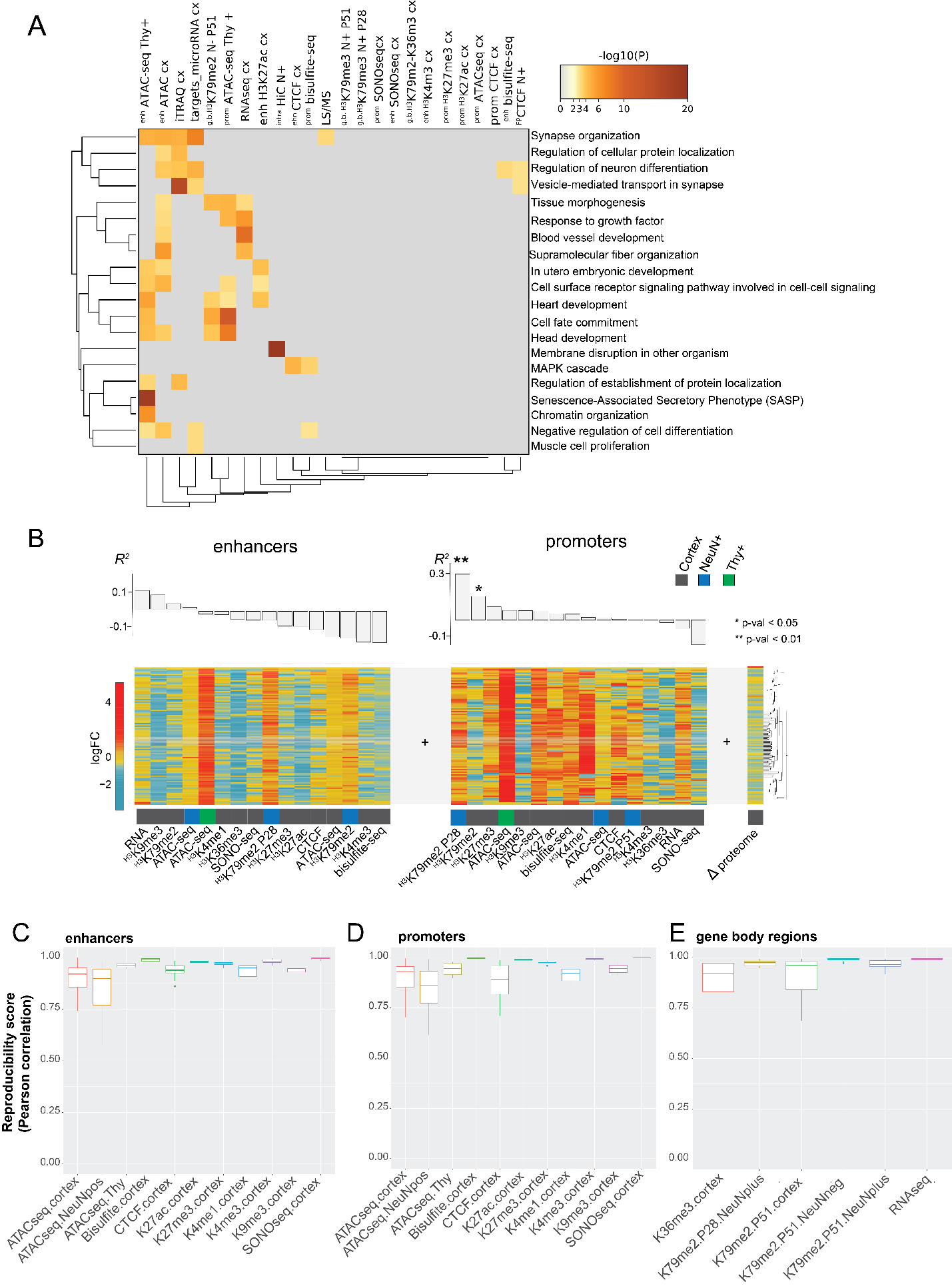


**Figure S7. Data integration and regulatory regions activity translated into proteomic changes. A)** Metascape gene ontology analysis of all differential changes induced by EE at FDR<0.05. **B)** Spearman translation efficiency of epigenetic marks in enhancers and promoters into differentially proteomic changes. **C, D, E)** Reproducibility score computed by pairwise Pearson correlations of biological replicates read counts in enhancers (C), promoters (D) and gene body regions (E).
